# Loss of HES-1 Expression Predicts a Poor Prognosis for Small Intestinal Adenocarcinoma Patients

**DOI:** 10.3389/fonc.2020.01427

**Published:** 2020-08-19

**Authors:** Jeong Won Kim, Sun-Young Jun, Kris Ylaya, Hee-Kyung Chang, Young-Ha Oh, Seung-Mo Hong, Joon-Yong Chung, Stephen M. Hewitt

**Affiliations:** ^1^Laboratory of Pathology, Center for Cancer Research, National Cancer Institute, National Institutes of Health, Bethesda, MD, United States; ^2^Department of Pathology, Kangnam Sacred Heart Hospital, Hallym University College of Medicine, Seoul, South Korea; ^3^Department of Pathology, Incheon St. Mary's Hospital, College of Medicine, The Catholic University of Korea, Seoul, South Korea; ^4^Department of Pathology, Kosin University College of Medicine, Busan, South Korea; ^5^Department of Pathology, Hanyang University College of Medicine, Seoul, South Korea; ^6^Department of Pathology, Asan Medical Center, University of Ulsan College of Medicine, Seoul, South Korea

**Keywords:** HES-1, *KRAS*, prognosis, small intestine, adenocarcinoma

## Abstract

**Objective:** Hairy and enhancer of split-1 (HES-1), which is a downstream target of the Notch signaling pathway, has been linked to *KRAS* mutations. HES-1 has been proposed as harboring oncogenic activity in colorectal cancer but has not been investigated in adenocarcinoma of the small intestine, where the drivers of oncogenesis are not as well-understood.

**Materials and Methods:** To investigate the clinicopathologic and prognostic implications of HES-1, HES-1 immunohistochemical expression was analyzed in digital images along with clinicopathological variables, including survival and *KRAS* genotype, in 185 small intestinal adenocarcinomas.

**Results:** The loss of HES-1 expression (HES-1^Loss^) was observed in 38.4% (71/185) of the patients, and was associated with higher pT category (*P* = 0.018), pancreatic invasion (*P* = 0.005), high grade (*P* = 0.043), and non-tubular histology (*P* = 0.004). Specifically, in tumors with mutant *KRAS* (*KRAS*^MT^), HES-1^Loss^ was related to proximal location (*P* = 0.024), high T and N categories (*P* = 0.005 and 0.047, respectively), and pancreatic invasion (*P* = 0.004). Patients with HES-1^Loss^ showed worse overall survival compared to those with intact HES-1 (HES-1^Intact^) (*P* = 0.013). Patients with HES-1^Loss^/*KRAS*^MT^ (median, 17.3 months) had significantly worse outcomes than those with HES-1^Intact^/*KRAS*^WT^ (39.9 months), HES-1^Intact^/*KRAS*^MT^ (47.6 month), and HES-1^Loss^/*KRAS*^WT^ (36.2 months; *P* = 0.010). By multivariate analysis, HES-1^Loss^ (hazard ratio = 1.55, 95% confidence interval (CI), 1.07–2.26; *P* = 0.022) remained an independent prognostic factor.

**Conclusion:** HES-1expression can be used as a potential prognostic marker and may aid in the management of patients with small intestinal adenocarcinomas.

## Introduction

Small intestinal adenocarcinoma is rare cancer that is clinically distinct from colorectal cancer but managed similarly due to the lack of prospective data necessary for establishing optimal management. However, recent studies demonstrated that the clinicopathologic and molecular features of small intestinal adenocarcinoma differed from those of colorectal cancer (1, 2). The majority of small intestinal adenocarcinoma patients are diagnosed at an advanced disease stage because of a lack of early detection tools, low incidence, and non-specific clinical symptoms (1, 3). A prospective ARCAD-NADEGE cohort study found that 35.6% of patients with small intestinal adenocarcinomas were diagnosed with metastatic disease, compared to 15.6% of those with colorectal cancer (4). The incidence of small intestinal adenocarcinoma is increasing while that of colorectal cancer is declining (5). Thus, there is an urgent need for research efforts on prognostic predictors and/or guides for the treatment of small intestinal adenocarcinoma.

The hairy and enhancer of split (HES) family of proteins consists of seven members that share a highly conserved tetrapeptide domain (Trp-Arg-Pro-Trp) at the C-terminus (6). Within this family, HES-1 is a transcriptional factor that plays an important role in intracellular processes, such as cell cycle arrest, differentiation, and apoptosis (7, 8). HES-1 is a downstream target of the Notch signaling pathway and is regulated by the Hedgehog and Wnt signaling pathways (9–11). It is expressed in the intestine along with HES-3, HES-5, HES-6, and HES-7 (12). Among the HESs, HES-1 is crucial for the normal development of the small intestine because it regulates the differentiation of Paneth cells (13). Prior studies suggested that HES-1 might play an oncogenic role in colorectal cancer. However, its role in tumorigenesis or prognosis remains unclear (11, 14–17). The clinicopathologic and prognostic significance of HES-1 expression has not been elucidated in small intestinal adenocarcinoma.

*KRAS* is the most frequently altered gene among the three human RAS isoforms and is mutated in approximately 30–50% of colorectal cancers (18–20). The significance of *KRAS* mutations has been demonstrated in a genetic colorectal cancer model, in which mutant *KRAS* harboring *adenomatous polyposis coli* (*APC*) mutations induced tumorigenesis and metastasis (21, 22). Schrock et al. (2) recently reported that *KRAS* was mutated in 53.6% (170/317) of the small intestinal adenocarcinomas. Abnormalities in *KRAS*-mediated differentiation and proliferation were linked to activation of the HES-1 transcription factor in colorectal carcinomas (23).

In this study, we investigated the clinicopathologic and prognostic significance of HES-1 expression in small intestinal adenocarcinomas by utilizing combined immunohistochemistry (IHC) and digital image analysis. In addition, we examined the potential clinical significance of HES-1 expression in patients with small intestinal adenocarcinomas harboring mutant *KRAS*.

## Materials and Methods

### Tissue Samples and Clinicopathological Data

This study was approved by the Institutional Review Board of Incheon St. Mary's Hospital (Seoul, Republic of Korea; OC14OIMI0133). A cohort of 197 patients who underwent surgical resections for primary small intestinal adenocarcinomas from the surgical pathology archives of 22 South Korean institutions were examined, as reported previously (24). Patients with primary carcinomas in the duodenum, jejunum, and ileum were included in the study. Patients with carcinomas grossly involving the stomach, the ampulla of Vater, pancreas, cecum, or appendix were excluded from the study.

The clinical and pathological data collected in a previous study were used in this study. The patient's gender, age, tumor location, and survival data were included as clinical data. Duodenal adenocarcinomas were defined as proximal tumors, whereas jejunal and/or ileal adenocarcinomas were considered distal tumors. Pathological data included histological type, differentiation, pathologic tumor-node-metastasis (pTNM) stage, lymph node metastasis, pancreatic invasion, and perineural and lymphovascular invasion. Histologic types and tumor grading were classified according to the 2019 World Health Organization (WHO) classification (25). All cases were staged according to the eighth edition of the American Joint Committee on Cancer (AJCC) cancer staging system (26).

### HES-1 Expression

Immunohistochemical staining was performed on tissue microarrays (TMAs), which were constructed as part of a previous study (27). Briefly, the representative areas of each sample were selected and marked on the corresponding hematoxylin and eosin-stained slides. Three tissue cylinders with 1-mm tumor diameter each and one matched core from normal mucosa were punched from each formalin-fixed, paraffin-embedded (FFPE) tissue block and transplanted into recipient blocks using a tissue arrayer (Beecher Instruments, Inc., Silver Spring, MD, USA).

For IHC, TMA sections with 5-μm thickness were deparaffinized in xylene and rehydrated in a graded ethanol series. The endogenous peroxidase activity of the samples was quenched with 3% H_2_O_2_ solution (Dako, Carpinteria, CA, USA) for 15 min at room temperature. Heat-induced antigen retrieval was performed for 20 min in a target retrieval buffer at pH 6.0 (Dako). The slides were then incubated with rabbit monoclonal anti-HES-1 antibody (Cell Signaling Technology, Danvers, MA, USA; clone D6P2U; cat# 11988) at 1:500 for 1 h at room temperature in a Dako Autostainer Plus Slide Stainer (Dako). Subsequently, the slides were incubated with Envision^+^Rb HRP dual-link secondary (Dako) and visualized with 3,3′-diaminobenzidine (Dako), and counterstained with hematoxylin. The primary antibody and rabbit immunoglobulin were omitted in the negative control, and human placenta was used as a positive control (28).

**Figure 1:**
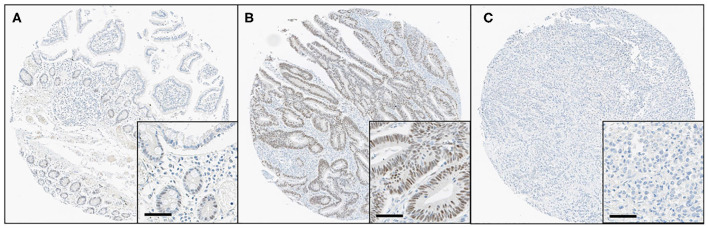
Immunohistochemical findings for HES-1 in small intestinal adenocarcinomas. **(A)** Some epithelial cells of basal crypts in normal mucosa show immunoreactivity for HES-1. **(B)** The tumor cells of low-grade small intestinal adenocarcinomas show diffuse, strong positivity for HES-1, corresponding to HES-1^Intact^. **(C)** In contrast, the high-grade tumor cells are completely negative for HES-1, indicating HES-1^Loss^ (Original magnification, ×8; inset, ×40; scale bar, 50 μm).

All immunostained slides were digitalized using an Aperio AT2 digital scanner (Leica Biosystems, Vista, CA, USA) at 40× objective magnification and the images were automatically analyzed using Visiopharm software v6.9.1 (Visiopharm, Hørsholm, Denmark). In brief, screenshots of single relevant regions of interest were generated by a single pathologist (JWK) who was blinded to the clinical and pathological data. Blue-colored (hematoxylin) tumor nuclei were initially defined, and then brown-colored (DAB) nuclei and cytoplasm were separated spectrally. Subsequently, the brown nuclear staining intensity (0 = negative, 1 = weak, 2 = moderate, and 3 = strong) and the percentage of nuclear-stained tumor cells (range, 0–100) were obtained using a predefined algorithm and optimized settings (Figure 1). Histoscores were calculated by multiplying the intensity score and proportion score and ranged from 0 to 300 (Supplementary Figure S1A). For the statistical analyses, the values were dichotomized using the cutoff value showing the most discriminative power. The samples with histoscores of 40.0 or lower were classified as loss of HES-1 expression (HES-1^Loss^), while cases with a histoscore higher than 40.0 were classified as intact HES-1 expression (HES-1^Intact^). There was no significant intra-tumor heterogeneity in HES-1 expression (Supplementary Figure S1B).

### KRAS Mutation

We used previously reported *KRAS* mutation data in the same cohort (29). In brief, genomic DNA was extracted from the FFPE tissue blocks by a QIAmp DNA Mini Kit (Qiagen, Valencia, CA, USA) as previously described (29). The *KRAS* genes were amplified using the following primers: forward, 5′-TGACATGTTCTAATATAGTCAC-3′, and reverse, 5′-ACAAGATTTACCTCTATTGTT-3′). The PCR reaction volume was 25 μl, including 0.3 μM of each primer and AmpliTaq Gold PCR Master Mix (Applied Biosystems, Foster City, CA, USA). The cycling conditions were as follows: initial denaturation at 95°C for 5 min, followed by 40 cycles of denaturing at 95°C for 50 s, annealing for 50 s, elongation at 72°C for 1 min, and a final elongation at 72°C for 7 min. The PCR amplicons were purified by a QIAquick PCR Purification Kit (Qiagen) and sequencing reactions were performed in the forward and reverse directions using the BigDye Terminator Cycle Sequencing Kit, version 1.1 (Applied Biosystems).

### Statistical Analysis

Unpaired Student's *t*-test was applied to compare the continuous variables. The relationships between the categorical variables were analyzed by the chi-squared test or Fisher's exact test. All survival analyses used an overall survival (OS) model, which captured all patient deaths as events and censored other patients at their last visit dates. The Kaplan–Meier method was used to compare survival between the groups and survival was analyzed by the log-rank test using a cutoff histoscore of 40.0. A Cox proportional hazards model was used to estimate the hazard ratios (HRs) and confidence intervals (CIs) in both the univariate and multivariate models. In all statistical analyses, a *P*-value of < 0.05 was considered statistically significant. Data analysis was performed using SPSS Statistics for Windows, version 23 (IBM Corp., Armonk, NY, USA).

## Results

### Clinicopathological Characteristics

The TMA contained 197 small intestinal adenocarcinoma samples. However, due to tissue loss and folding during sectioning and staining, along with sample heterogeneity, only 185 samples could be interpreted and included in this study. One hundred sixteen patients were male (62.7%) and 69 were female (37.3%), with a mean age of 58.9 years (range, 23 to 86 years). The most common tumor location was the duodenum in 103 (55.7%) patients, followed by the jejunum in 54 (29.2%), and the ileum in 28 (15.1%) patients. The clinicopathological characteristics of the study are summarized in Supplementary Table S1. The patients were followed-up for a median of 28.8 months, ranging from 0.3 to 168.4 months.

### HES-1 Expression

The histoscores of the nuclear HES-1 expression ranged from 0 to 290.7, with a median of 62.2. Of the cancer specimens, 114 (61.6%) of the185 cases exhibited HES-1^Intact^, whereas 71 (38.4%) cases showed HES-1^Loss^. As summarized in Table 1, HES-1^Loss^ was significantly associated with younger age (<60 years; *P* = 0.023). In terms of histologic subtype, non-tubular adenocarcinomas, including mucinous, signet ring cell, and undifferentiated carcinomas, frequently showed HES-1^Loss^, whereas tubular adenocarcinomas tended to have HES-1^Intact^ (*P* = 0.004). HES-1^Loss^ was more frequent in carcinomas with extended T category (*P* = 0.018), high grade (*P* = 0.043), and pancreatic invasion (*P* = 0.005). No significant association was identified between HES-1 expression and other clinicopathological variables, including sex, tumor location, type of growth, lymphovascular and perineural invasion, pN category, stage group, and *KRAS* genotype.

**Table 1:** Correlation between clinicopathologic factors and HES-1 expression of small intestinal adenocarcinoma patients.

**Category (No, %)**	**HES-1^**Intact**^**	**HES-1^**Loss**^**	***P-*value**
**Age**			0.023^*^
<60 years	51 (44.7)	44 (62.0)	
≥60 years	63 (55.3)	27 (38.0)	
**Sex**			0.161
Male	67 (58.8)	49 (69.0)	
Female	47 (41.2)	22 (31.0)	
**Location**			0.174
Proximal (duodenum)	59 (51.8)	44 (62.0)	
Distal (jejunum and ileum)	55 (48.2)	27 (38.0)	
**Growth pattern^a^**			0.170
Polypoid and nodular	23 (21.3)	21 (30.4)	
Infiltrative	85 (78.7)	48 (69.6)	
**Histological subtype**			0.004^*^
Tubular adenocarcinoma	109 (95.6)	59 (83.1)	
Non-tubular carcinoma^b^	5 (4.4)	12 (16.9)	
**Grade**			0.043^*^
Low (well and moderately differentiated)	92 (80.7)	48 (67.6)	
High (poorly differentiated and undifferentiated)	22 (19.3)	23 (32.4)	
**Lymphovascular invasion**			0.208
Absent	59 (51.8)	30 (42.3)	
Present	55 (48.2)	41 (57.7)	
**Pancreatic invasion**			0.005^*^
Absent	81 (71.1)	36 (50.7)	
Present	33 (28.9)	35 (49.3)	
**Perineural invasion**			0.835
Absent	77 (67.5)	49 (69.0)	
Present	37 (32.5)	22 (31.0)	
**pT category**			0.018^*^
pT_is_-pT_2_	16 (14.0)	3 (4.2)	
pT_3_	41 (36.0)	19 (26.8)	
pT_4_	57 (50.0)	49 (69.0)	
**pN category^c^**			0.175
pN_0_	54 (52.4)	28 (41.8)	
pN_1_+pN_2_	49 (47.6)	39 (58.2)	
**Stage group^c^**			0.185
0–I	12 (11.6)	3 (4.5)	
II	42 (40.8)	25 (37.3)	
III	49 (47.6)	39 (58.2)	
***KRAS*** **genotype**			0.108
*KRAS*^WT^	82 (71.9)	43 (60.6)	
*KRAS*^MT^	32 (28.1)	28 (39.4)	

* *Statistically significant (P < 0.05)*.

a *Calculated with only 177 cases with available information on growth type*.

b *The non-tubular types included mucinous carcinomas (n = 9), signet ring cell carcinomas (n = 4), and undifferentiated carcinoma (n = 4)*.

c *Calculated with only 170 cases with available information on lymph node metastasis and stage grouping*.

### KRAS Mutation

**Figure 2:**
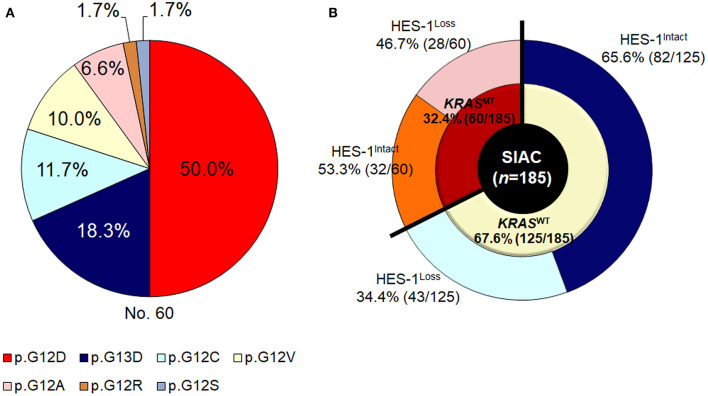
*KRAS* genotypes of small intestinal adenocarcinomas. **(A)** Frequency of *KRAS*^MT^ and **(B)** HES-1 expression according to *KRAS* genotypes.

*KRAS* mutations (*KRAS*^MT^) were found in 32.4% (60/185) of the patients. Among the small intestinal adenocarcinomas with *KRAS*^MT^, 81.7% (49/60) of the mutations were detected in codon 12 and 18.3% (11/60) were identified in codon 13. The main type of *KRAS*^MT^ was p.G12D (30/60 cases, 50.0%), followed by p.G13D (11/60, 18.3%), p.G12C (7/60, 11.7%), p.G12V (6/60, 10.0%), p.G12A (4/60, 6.6%), p.G12R (1/60, 1.7%), and p.G12S (1/60, 1.7%) (Figure 2A).

### HES-1 Expression and KRAS Genotypes

As described in Figure 2B, in the *KRAS*^WT^ group (*n* = 125), 43 (34.4%) cases exhibited HES-1^Loss^. In contrast, in tumors with *KRAS*^MT^ (*n* = 60), 28 (46.8%) had HES-1^Loss^. The relationship between HES-1 expression and clinicopathologic factors according to *KRAS* mutation status are summarized in Table 2. In the *KRAS*^MT^ group, HES-1^Loss^ was significantly associated with higher pT category (*P* = 0.005), proximal location (*P* = 0.024), pancreatic invasion (*P* = 0.004), and nodal metastasis (*P* = 0.047). In the *KRAS*^WT^ group, HES-1^Loss^ was only correlated with non-tubular types of small intestinal adenocarcinomas (*P* = 0.028).

**Table 2:** Correlation between clinicopathologic factors and HES-1 expression based on *KRAS* genotype in small intestinal adenocarcinoma patients.

**Category (No, %)**	***KRAS*** **genotype**					
	***KRAS***^****WT****^			***KRAS***^****MT****^		
	**HES-1^**Intact**^**	**HES-1^**Loss**^**	***P***	**HES-1^**Intact**^**	**HES-1^**Loss**^**	***P***
**Age**			0.287			0.064
<60 years	38 (46.3)	25 (58.1)		13 (40.6)	19 (67.9)	
≥60 years	44 (53.7)	18 (41.9)		19 (59.4)	9 (32.1)	
**Sex**			0.091			1.000
Male	47 (57.3)	32 (74.4)		20 (62.5)	17 (60.7)	
Female	35 (42.7)	11 (25.6)		12 (37.5)	11 (39.3)	
**Tumor location**			0.939			0.024^*^
Proximal	44 (53.7)	22 (51.2)		15 (46.9)	22 (78.6)	
Distal	38 (46.3)	21 (48.8)		17 (53.1)	6 (21.4)	
**Growth pattern^a^**			0.231			0.835
Polypoid and nodular	17 (22.1)	14 (34.1)		6 (19.4)	7 (25.0)	
Infiltrative	60 (77.9)	27 (65.9)		25 (80.6)	21 (75.0)	
**Histological subtype**			0.028^*^			0.192
Tubular adenocarcinoma	77 (93.9)	34 (79.1)		32 (100.0)	25 (89.3)	
Non-tubular carcinoma^b^	5 (6.1)	9 (20.9)		0 (0.0)	3 (10.7)	
**Grade**			0.087			0.346
Low (well and moderately differentiated)	63 (76.8)	26 (60.5)		29 (90.6)	22 (78.6)	
High (poorly differentiated and undifferentiated)	19 (23.2)	17 (39.5)		3 (9.4)	6 (21.4)	
**Lymphovascular invasion**			1.000			0.070
Absent	39 (47.6)	20 (46.5)		20 (62.5)	10 (35.7)	
Present	43 (52.4)	23 (53.5)		12 (37.5)	18 (64.3)	
**Pancreatic invasion**			0.559			0.004^*^
Absent	59 (72.0)	28 (65.1)		22 (68.8)	8 (28.6)	
Present	23 (28.0)	15 (34.9)		10 (31.2)	20 (71.4)	
**Perineural invasion**			0.916			1.000
Absent	55 (67.1)	30 (69.8)		22 (68.8)	19 (67.9)	
Present	27 (32.9)	13 (30.2)		10 (31.2)	9 (32.1)	
**pT category**			0.312			0.005^*^
pT_is_-pT_2_	11 (13.4)	2 (4.7)		5 (15.6)	1 (3.6)	
pT_3_	30 (36.6)	17 (39.5)		11 (34.4)	2 (7.1)	
pT_4_	41 (50.0)	24 (55.8)		16 (50.0)	25 (89.3)	
**pN category^a^**			1.000			0.047^*^
pN_0_	35 (47.3)	18 (46.2)		19 (65.5)	10 (35.7)	
pN_1_+pN_2_	39 (52.7)	21 (53.8)		10 (34.5)	18 (64.3)	
**Stage group^a^**			0.584			0.062
0–I	8 (10.8)	2 (5.1)		4 (13.8)	1 (3.6)	
II	27 (36.5)	16 (41.0)		15 (51.7)	9 (32.1)	
III	39 (52.7)	21 (53.9)		10 (34.5)	18 (64.3)	

* *Statistically significant (P < 0.05)*.

a *Calculated using only cases with available information*.

b *The non-tubular types included mucinous carcinomas (n = 9), signet ring cell carcinomas (n = 4), and undifferentiated carcinoma (n = 4)*.

### Survival Analysis

**Figure 3:**
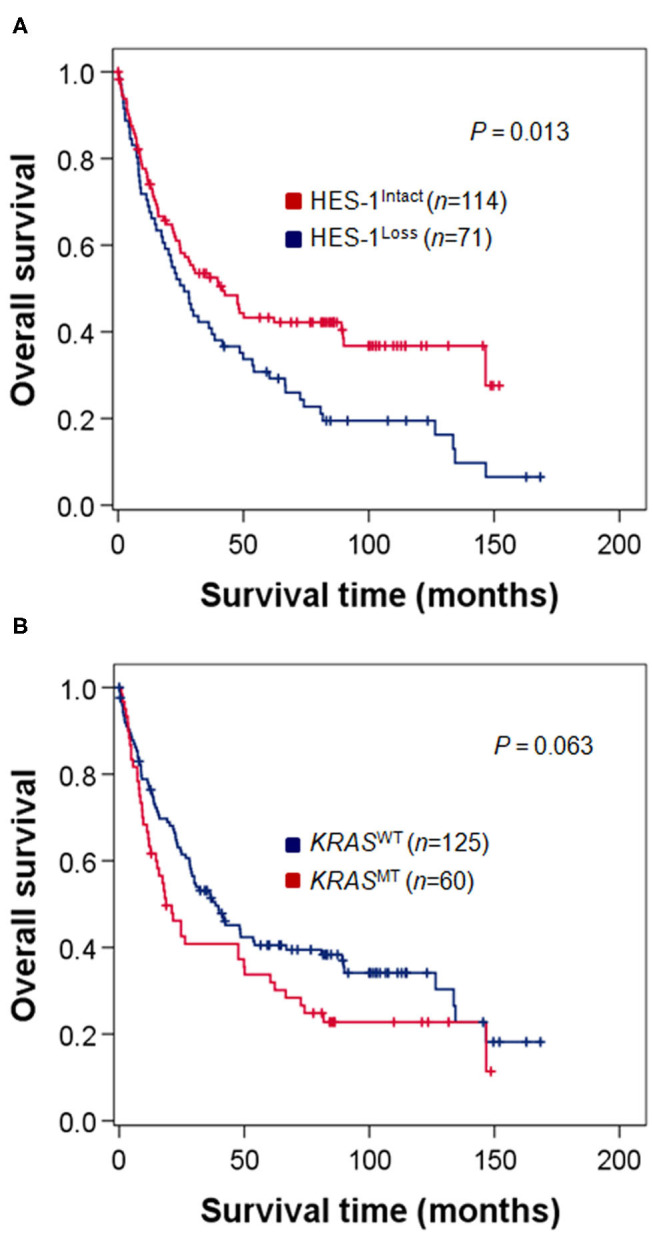
Survival analysis of patients with small intestinal adenocarcinomas. **(A)** Patients with HES-1^Loss^ show poor OS compared to those with HES-1^Intact^ (median, 26.3 vs. 41.7 months, *P* = 0.013). **(B)** Patients with *KRAS*^MT^ exhibit a tendency toward worse OS than those with *KRAS*^WT^, but the difference was not statistically significant (18.7 vs. 38.5 months, *P* = 0.063).

The relationship between HES-1 expression and OS is described in Figure 3. Patients with HES-1^Loss^ (median, 26.3 months) had significantly shorter OS times than those with HES-1^Intact^ (41.7 months; *P* = 0.013) (Figure 3A). The median OS of the patients with *KRAS*^MT^ tended to be shorter than that of the patients with *KRAS*^WT^ (18.7 vs. 38.5 months), but it did not reach statistical significance (*P* = 0.063, Figure 3B). In the *KRAS*^MT^ subgroup, patients with HES-1^Loss^ (median, 17.3 months) had worse OS than those with HES-1^Intact^ (47.6 months; *P* = 0.027), whereas there was no significant survival difference in the *KRAS*^WT^ subgroup based on HES-1 expression status (Supplementary Figure S2).

### Survival Analysis Based on HES-1 Expression and KRAS Genotypes

**Figure 4:**
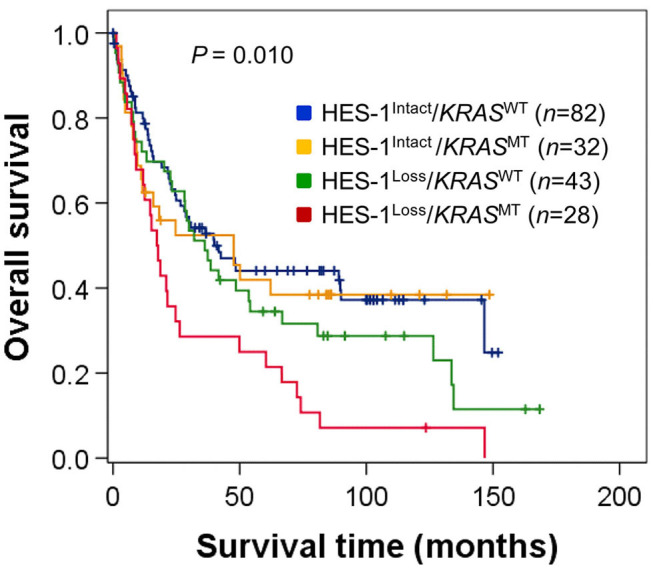
Survival analysis of patients with small intestinal adenocarcinomas according to the combined patterns of HES-1 expression and *KRAS* genotypes. Survival differences are observed among four groups classified according to HES-1 expression and *KRAS* genotype (log-rank, *P* = 0.010).

Furthermore, we analyzed the OS of patients in the four groups, which were classified according to the combined patterns of HES-1 expression and *KRAS* genotypes: HES-1^Loss^/*KRAS*^MT^ (28 cases, 15.2%), HES-1^Loss^/*KRAS*^WT^ (43, 23.2%), HES-1^Intact^/*KRAS*^MT^ (32, 17.3%), and HES-1^Intact^/*KRAS*^WT^ (82, 44.3%). Patients with HES-1^Loss^/*KRAS*^MT^ (median, 17.3 months) had significantly worse outcomes than those with HES-1^Intact^/*KRAS*^WT^ (39.9 months), HES-1^Intact^/*KRAS*^MT^ (47.6 month), and HES-1^Loss^/*KRAS*^WT^ (36.2 months) (*P* = 0.010; Figure 4). Significant differences in survival rates were observed between the groups with HES-1^Loss^/*KRAS*^MT^ and HES-1^Intact^/*KRAS*^WT^ (*P* = 0.001), and HES-1^Loss^/*KRAS*^WT^ and HES-1^Loss^/*KRAS*^MT^ (*P* = 0.001) in pair-wise comparisons. However, there were no significant differences between the HES-1^Intact^/*KRAS*^WT^ and HES-1^Intact^/*KRAS*^MT^ (*P* = 0.252), and HES-1^Intact^/*KRAS*^MT^ and HES-1^Loss^/*KRAS*^WT^ (*P* = 0.533) groups.

### Multivariate Analysis

Cox multivariate proportional hazard analyses were performed using HES-1 expression and other factors considered significant by univariate analysis (Table 3). Non-tubular histology (HR = 1.771, 95% CI: 0.994–3.157, *P* = 0.053) and *KRAS*^MT^ (HR = 1.410, 95% CI: 0.980–2.030, *P* = 0.064) were exhibited a tendency of shorter patient survival by univariate survival analysis. Distal location (HR = 1.291, 95% CI: 1.055–1.580, *P* = 0.013), high pT category (≥pT_3_) (HR = 1.380, 95% CI: 1.039–1.834, *P* = 0.026), lymph node metastasis (HR = 1.813, 95% CI: 1.221–2.691, *P* = 0.003), and HES-1^Loss^ (HR = 1.551, 95% CI: 1.067–2.255, *P* = 0.022) were revealed as independent negative prognostic factors. Notably, dual HES-1^Loss^ and *KRAS*^MT^ is also an independent prognostic factor for poor OS in small intestinal adenocarcinoma patients (HR = 1.312 [95% CI: 1.125–1.529], *P* = 0.001; Supplementary Table S2).

**Table 3:** Univariate and multivariate analyses of OS in small intestinal adenocarcinoma patients.

**Variables**	**Univariate analysis**		**Multivariate analysis**	
	**HR [95% CI]**	***P***	**HR [95% CI]**	***P***
Age (≥ 60 years)	1.220 [0.860–1.730]	0.274		
Sex (female)	1.110 [0.780–1.600]	0.557		
Location (distal)	1.280 [1.070–1.530]	0.007^*^	1.291 [1.055–1.580]	0.013^*^
Histologic subtype	1.771 [0.994–3.157]	0.053		
(non-tubular)				
Grade (high)	1.240 [0.840–1.850]	0.280		
pT category (≥ pT_3_)	1.460 [1.160–1.840]	0.001^*^	1.380 [1.039–1.834]	0.026^*^
Nodal metastasis	2.160 [1.470–3.170]	<0.001^*^	1.813 [1.221–2.691]	0.003^*^
Pancreatic invasion	0.860 [0.600–1.230]	0.403		
Perineural invasion	1.380 [0.950–2.010]	0.090		
*KRAS*^MT^	1.410 [0.980–2.030]	0.064		
HES-1^Loss^	1.550 [1.109–2.200]	0.014^*^	1.551 [1.067–2.255]	0.022^*^

*HR, Hazard ratio; CI, confidence interval*.

* *Statistically significant (P < 0.05)*.

## Discussion

Notch signaling not only affects cell differentiation, proliferation, and apoptosis but controls the expression of HES-1 (30, 31). In general, Notch signaling is known to suppress squamous cancers of the skin, but stimulate hematologic malignancies and adenocarcinomas of the stomach, colon, and pancreas (30). A previous study has reported that Notch3 expression is correlated with lower T stage and the absence of lymphovascular invasion in small intestinal adenocarcinomas (32). HES-1 is known as a transcriptional inhibitor. However, recent studies showed that HES-1 was more than a repressor and contributed to cancer stem cell maintenance, cancer metastasis, and tumor multidrug resistance (33). The regulation of HES-1 expression is mediated by not only the canonical Notch signaling pathway, but also other signaling pathways, such as Hedgehog, c-Jun N-terminal kinase, Wnt, and TGF-a/Ras/mitogen-activated protein kinase (MAPK) (10, 33, 34). To our knowledge, this is the first study to assess the prognostic value of HES-1 expression, alone and in combination with the *KRAS* genotype in patients with small intestinal adenocarcinomas.

We found HES-1^Loss^ to be strongly associated with tumor aggressiveness, indicated by high T category, high grade, pancreatic invasion, and carcinoma showing non-tubular histology. Moreover, HES-1^Loss^ was an independent poor prognostic factor of small intestinal adenocarcinomas for OS. In contrast to our results, some studies have demonstrated that increased HES-1 expression may be an adverse prognostic factor in colorectal cancer (15, 35). We noted that those studies evaluated HES-1 expression via mRNA rather than IHC. Since HES-1 expression is sometimes preserved in non-neoplastic stromal cells of the colorectal mucosa, mRNA expression assays would have measured both tumor and stromal HES-1 expression (16). To accurately assess the nuclear expression of HES-1 in tumor cells via IHC, we selected regions only composed of tumor cell nests and analyzed them using digital image analysis. Consistent with our findings, a recent study performed by Ahadi et al. reported that the loss of HES-1 nuclear expression in colorectal carcinomas was significantly associated with mucinous or medullary histology, higher histological grade, and worse survival (16). With regards to tumor histology, Vanoli et al. demonstrated that non-glandular histology type is an independent prognostic factor for poor outcome in small intestinal adenocarcinoma patients (36). In this study, patients with non-tubular small intestinal adenocarcinomas had a tendency of worse OS, but it was not statistically significant (Table 3). This discrepancy might be due to the small proportion of non-tubular type of tumors (9.2%, 17/185) in this study, comparing to higher proportion of them (44.7%, 34/76) in the study of Vanoli et al. Further studies utilizing large numbers of non-tubular type of small intestinal adenocarcinomas are needed to establish the prognostic power of different histologic features.

It has been hypothesized that the pathogenesis of small intestinal adenocarcinoma varies depending upon the tumor location (4). Proximal small intestinal carcinomas are sometimes accompanied by background gastric metaplasia, suggesting a gastric metaplasia-dysplasia-carcinoma sequence, or pancreaticobiliary differentiation. In contrast, distal small intestine carcinomas are significantly associated with Crohn's disease (4).

Studies on the interactions between Notch signaling, HES-1 expression, and *KRAS* mutations in gastrointestinal cancers have been limited and contradictory. Nishikawa et al. (37) suggested that mutant *KRAS*-induced HES-1 played an essential role in the initiation and progression of pancreatic ductal adenocarcinoma by regulating acinar-to-ductal reprogramming-related genes. Feng et al. (23) also reported that in colorectal carcinomas, abnormalities in *KRAS*-mediated differentiation and proliferation required MAPK signaling and were linked to activation of the HES-1 transcription factor. Meanwhile, Chung et al. (38) suggested that downregulation of Notch signaling occurs during the initiation of *KRAS*-driven gastric carcinogenesis. Based on these findings, we hypothesized that the *KRAS* mutation status and HES-1 expression may be associated with tumor location in small intestinal adenocarcinomas, and Notch-independent HES-1 expression may be linked to mutated *KRAS*. In this study, we found that the survival rates of patients with *KRAS*^MT^ were significantly reduced only in the loss of HES-1 expression (HES-1^Loss^/*KRAS*^WT^ and HES-1^Loss^/*KRAS*^MT^, *P* = 0.001), while the survival rate of patients with HES-1^Intact^ tumors was not dependent on *KRAS* mutation status (HES-1^Intact^/*KRAS*^WT^ and HES-1^Intact^/*KRAS*^MT^, *P* = 0.252) (Figure 4). Thus, we investigated HES-1^Loss^/*KRAS*^MT^ and found that it could be an independent prognostic marker for poor OS time in small intestinal adenocarcinoma patients (Supplementary Table S2). HES-1^Loss^ harboring *KRAS*^MT^ was more frequently detected in proximal location compared to distal location, which suggests a link between HES-1^Loss^/*KRAS*^MT^ and tumor location. These findings suggest that intact HES-1 expression, independent of *KRAS* genotype, prolonged the survival rate of small intestinal adenocarcinoma patients. Furthermore, HES-1^Loss^ was revealed as an independent prognostic factor for poor outcome. Further studies are needed to clarify the relationship between HES-1 and *KRAS* genotype in small intestinal adenocarcinomas.

The Notch signaling pathway is a promising target for anti-cancer therapy (30). However, activation of this pathway can lead to tumor-suppressive or oncogenic effects, and nonspecific inhibition of the Notch pathways has been toxic (30). In metastatic colon cancer, a phase II clinical trial of RO-4929097 targeting cleavage mediated by γ-secretase, which is a crucial step in Notch activation, was evaluated. However, there was no evidence of objective radiographic response and survival increase (39). As γ-secretase inhibitor (GSI) nonspecifically inhibits the Notch target gene, it causes a rapid differentiation of intestinal progenitor cells into goblet cells and this may be the primary cause of gastrointestinal toxicities associated with GSI (33, 40). Therefore, aiming at HES-1 may result in fewer side effects because many other Notch target genes will be unaffected (33). Moreover, since HES-1 lies at the crossroads of multiple signaling pathways, the co-inhibition of these pathways through targeting HES-1 might represent a new strategy for cancer therapy (33). It is also notable that the regulation of HES-1 expression and Notch pathway activity is dependent upon tissue, spatial, and temporal factors and the proteins with which they interact (10, 34). Therefore, we proposed that a more sophisticated approach is needed for tailored therapy targeting the Notch pathway in small intestinal adenocarcinomas.

Akce et al. demonstrated that duodenal localization tends to have worse patients' survival than jejunal/ileal adenocarcinomas (1). In contrast, we found that patients with distal (jejunal/ileal) adenocarcinomas had significantly shorter OS times than those with proximal (duodenal) adenocarcinomas. The present study only included surgically resected cases without stage IV disease, while the study by Akce et al. using a cohort derived from the National Cancer Data Base (*n* = 7,954) included inoperable stage IV cases (*n* = 2,889) (1). In addition, in the study of Akce et al., 37.6% of patients had duodenal adenocarcinomas presented as stage IV disease. Thus, this discrepancy may be resulted from differences in percentage of patients with surgical resection, ethnicity, and lifestyle.

In this study, we revealed that HES-1^Loss^ was associated with tumor aggressiveness, including high T category, high grade, and pancreatic invasion in small intestinal adenocarcinomas. Moreover, HES-1^Loss^ could predict a worse prognosis in patients with small intestinal adenocarcinomas. Further elucidation of the underlying molecular mechanism of HES-1 will contribute to development of new therapeutic targets in patients with small intestinal adenocarcinomas.

## Data Availability Statement

The raw data supporting the conclusions of this article will be made available by the authors, without undue reservation.

## Ethics Statement

The studies involving human participants were reviewed and approved by the Institutional Review Board of Incheon St. Mary's Hospital (OC14OIMI0133). The patients/participants provided their written informed consent to participate in this study.

## Author Contributions

JK, S-YJ, J-YC, and SH designed the study. JK, S-YJ, KY, H-KC, Y-HO, S-MH, and J-YC collected the experimental or clinical data. JK, J-YC, and SH analyzed the data. JK, S-YJ, and J-YC drafted and edited the manuscript. J-YC and SH reviewed and edited the manuscript. All authors read and approved the final manuscript.

## Conflict of Interest

The authors declare that the research was conducted in the absence of any commercial or financial relationships that could be construed as a potential conflict of interest.
