# Supplementary material for: Loss of HES-1 Expression Predicts a Poor Prognosis for Small Intestinal Adenocarcinoma Patients
Source: Front Oncol. 2020 Aug 19;10:1427. doi: 10.3389/fonc.2020.01427 (PMC7466551; doi:10.3389/fonc.2020.01427)
Supplement: Supplementary file 1 [file Table_1.docx]

**Supplementary Table S1.** Clinicopathologic characteristics of patients with small intestinal adenocarcinomas

| **Category (N = 185)** | **No. (*%*)** |
| --- | --- |
| **Age** |  |
| < 60 years | 95 (51.4) |
| ≥ 60 years | 90 (48.6) |
| **Sex** |  |
| Male | 116 (62.7) |
| Female | 69 (37.3) |
| **Location** |  |
| Duodenum | 103 (55.7) |
| Jejunum | 54 (29.2) |
| Ileum | 28 (15.1) |
| **Type of growtha** |  |
| Polypoid | 33 (18.7) |
| Nodular | 11 (6.2) |
| Infiltrative | 133 (75.1) |
| **Histological subtype** |  |
| Adenocarcinoma | 168 (90.8) |
| Mucinous carcinoma | 9 (4.8) |
| Signet ring cell carcinoma | 4 (2.2) |
| Undifferentiated carcinoma | 4 (2.2) |
| **Grade** |  |
| Low | 140 (75.7) |
| High | 45 (24.3) |
| **Lymphovascular invasion** |  |
| Absent | 89 (48.1) |
| Present | 96 (51.9) |
| **Perineural invasion** |  |
| Absent | 126 (68.1) |
| Present | 59 (31.9) |
| **Pancreatic invasion** |  |
| Absent | 117 (63.2) |
| Present | 68 (36.8) |
| **pT category** |  |
| pTis | 3 (1.6) |
| pT1 | 7 (3.8) |
| pT2 | 9 (4.9) |
| pT3 | 60 (32.4) |
| pT4 | 106 (57.3) |
| **pN categoryb** |  |
| pN0 | 82 (48.2) |
| pN1 | 43 (25.3) |
| pN2 | 45 (26.5) |
| **Stage groupb** |  |

| 0 | 2 (1.2) |
| --- | --- |
| I | 13 (7.6) |
| II | 67 (39.4) |
| III | 88 (51.8) |
| ***KRAS* genotype** |  |
| *KRAS*WT | 125 (67.6) |
| *KRAS*MT | 60 (32.4) |

aCalculated for only 177 cases with available information regarding growth type.

bCalculated for only 170 cases with available information regarding lymph node metastasis and stage grouping.

**Supplementary Table S2.** Cox proportional univariate and multivariate analyses of OS in small intestinal adenocarcinoma patients

| **Variables** | **Univariate analysis** |  |  | **Multivariate analysis** |  |
| --- | --- | --- | --- | --- | --- |
|  | **HR [95% CI]** | ***P*** |  | **HR [95% CI]** | ***P*** |
| Age (≥ 60 years) | 1.220 [0.860-1.730] | 0.274 |  |  |  |
| Sex (female) | 1.110 [0.780-1.600] | 0.557 |  |  |  |
| Location (distal)  Histologic subtype (nontubular) | 1.280 [1.070-1.530]  1.771 [0.994-3.157] | 0.007*  0.053 |  | 1.340 [1.093-1.643] | 0.005* |
| Grade (high) | 1.240 [0.840-1.850] | 0.280 |  |  |  |
| pT category (≥ pT3) | 1.460 [1.160-1.840] | 0.001* |  | 1.311 [0.996-1.726] | 0.053 |
| Nodal metastasis | 2.160 [1.470-3.170] | <0.001* |  | 1.882 [1.266-2.798] | 0.002* |
| Pancreatic invasion | 0.860 [0.600-1.230] | 0.403 |  |  |  |
| Perineural invasion | 1.380 [0.950-2.010] | 0.090 |  |  |  |
| Dual HES-1Loss and *KRAS*MT | 1.550 [1.109-2.200] | 0.014* |  | 1.312 [1.125-1.529] | 0.001* |

HR, Hazard ratio; CI, confidence interval

*Statistically significant (*P* < 0.05)
