# Supplementary material for: Loss of HES-1 Expression Predicts a Poor Prognosis for Small Intestinal Adenocarcinoma Patients
Source: Front Oncol. 2020 Aug 19;10:1427. doi: 10.3389/fonc.2020.01427 (PMC7466551; doi:10.3389/fonc.2020.01427)
Supplement: Supplementary file 2 [file Data_Sheet_1.pdf]

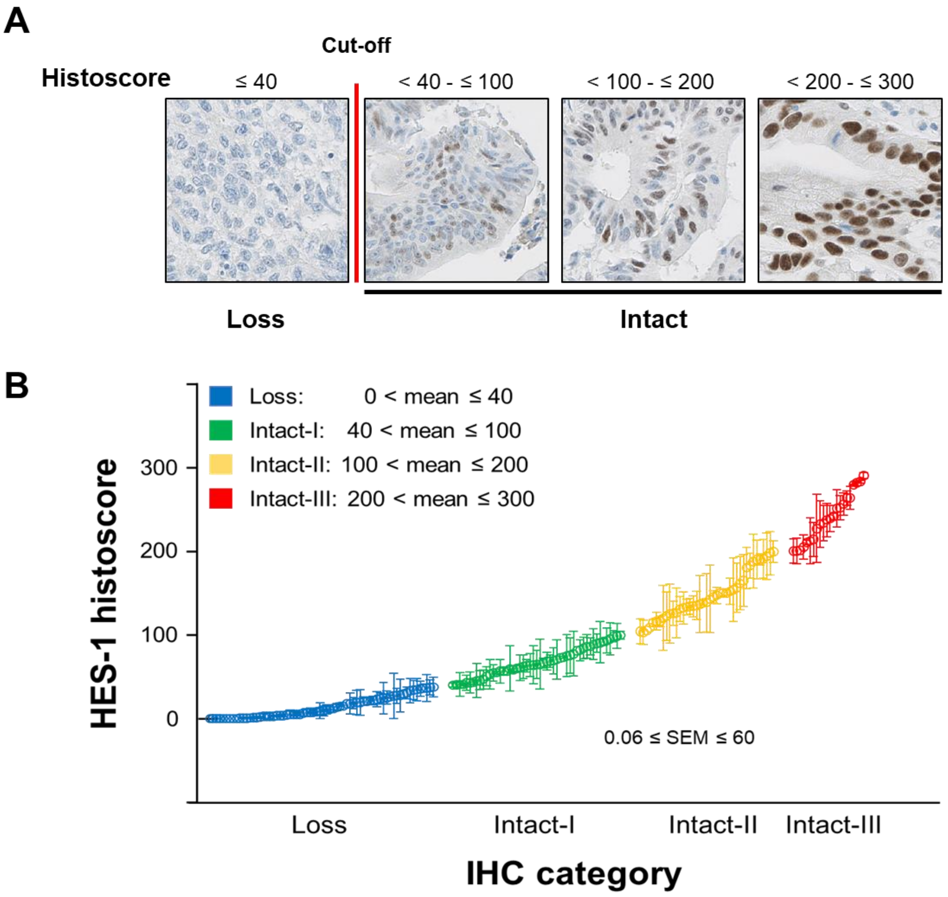

Supplementary Figure S1. HES-1 expression in small intestinal adenocarcinomas. (**A**) Representative staining images was displayed based on histoscore category. (**B**) There was no significant intra-tumor heterogeneity in small intestinal adenocarcinomas. Data represent the mean ± SEM.

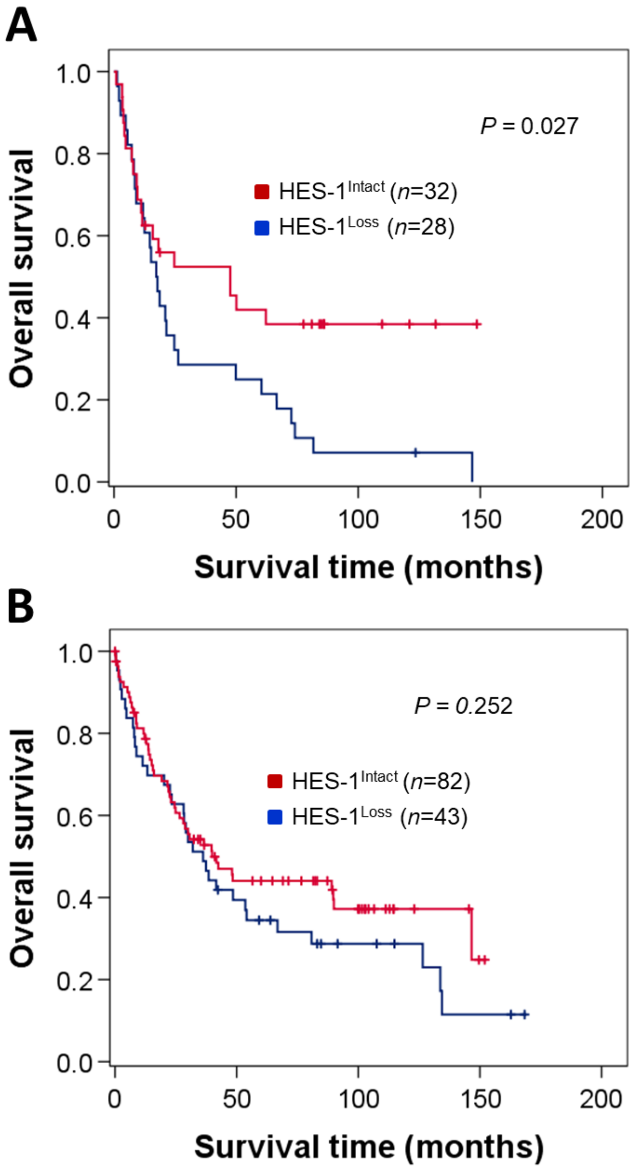

Supplementary Figure S2. Survival analysis of patients with small intestinal adenocarcinomas according to *KRAS* genotypes. **(A)** In the *KRAS*^{MT} group, patients with HES-1^{Loss} (median, 17.3 months; OS rate, 3.6%)

had worse OS than those with HES-1^{Intact} (47.6 months; 40.6%) (*P* = 0.027), whereas **(B)** there was no significant survival difference in the *KRAS*^{WT} group based on the HES-1 expression status (*P* = 0.252).
